# Differential chemosensitization of P-glycoprotein overexpressing K562/Adr cells by withaferin A and Siamois polyphenols

**DOI:** 10.1186/1476-4598-9-99

**Published:** 2010-05-03

**Authors:** Wipob Suttana, Samlee Mankhetkorn, Wilart Poompimon, Ajay Palagani, Sergey Zhokhov, Sarah Gerlo, Guy Haegeman, Wim Vanden Berghe

**Affiliations:** 1 Laboratory of Physical Chemistry, Molecular and Cellular Biology and Center of Excellence for Molecular Imaging, Department of Radiologic Technology, Faculty of Associated Medical Sciences, Chiang Mai University, Chiang Mai 50200, Thailand; 2 Laboratory of Natural Products, Department of Chemistry, Faculty of Science, Lampang Rajabhat University, Lampang, Thailand; 3 Laboratory of Eukaryotic Gene Expression and Signal Transduction (LEGEST), Department of Physiology, Ghent University, K.L.Ledeganckstraat 35, Gent, Belgium; 4 Lab Protein Science, Proteomics and Epigenetic Signaling, Department of Biomedical Sciences, University Antwerp, Campus Drie Eiken, Universiteitsplein 1, Wilrijk, Belgium

## Abstract

**Background:**

Multidrug resistance (MDR) is a major obstacle in cancer treatment and is often the result of overexpression of the drug efflux protein, P-glycoprotein (P-gp), as a consequence of hyperactivation of NFκB, AP1 and Nrf2 transcription factors. In addition to effluxing chemotherapeutic drugs, P-gp also plays a specific role in blocking caspase-dependent apoptotic pathways. One feature that cytotoxic treatments of cancer have in common is activation of the transcription factor NFκB, which regulates inflammation, cell survival and P-gp expression and suppresses the apoptotic potential of chemotherapeutic agents. As such, NFκB inhibitors may promote apoptosis in cancer cells and could be used to overcome resistance to chemotherapeutic agents.

**Results:**

Although the natural withanolide withaferin A and polyphenol quercetin, show comparable inhibition of NFκB target genes (involved in inflammation, angiogenesis, cell cycle, metastasis, anti-apoptosis and multidrug resistance) in doxorubicin-sensitive K562 and -resistant K562/Adr cells, only withaferin A can overcome attenuated caspase activation and apoptosis in K562/Adr cells, whereas quercetin-dependent caspase activation and apoptosis is delayed only. Interestingly, although withaferin A and quercetin treatments both decrease intracellular protein levels of Bcl2, Bim and P-Bad, only withaferin A decreases protein levels of cytoskeletal tubulin, concomitantly with potent PARP cleavage, caspase 3 activation and apoptosis, at least in part via a direct thiol oxidation mechanism.

**Conclusions:**

This demonstrates that different classes of natural NFκB inhibitors can show different chemosensitizing effects in P-gp overexpressing cancer cells with impaired caspase activation and attenuated apoptosis.

## Background

The cytotoxicity of chemotherapeutic agents is attributed to apoptosis. One feature that cytotoxic treatments of cancer have in common is their activation of the transcription factor NFκB, which regulates cell survival, suppresses the apoptotic potential of chemotherapeutic agents and contributes to drug resistance [1]. Acquired resistance to the effects of chemotherapy has emerged as a significant impediment to effective cancer therapy. As such, it is believed that inhibitors of NFκB might promote apoptosis in cancer cells and can be helpful to overcome resistance to chemotherapeutic agents.

Nuclear factor kappa B (NFκB) is a family of transcription factors that play important roles in regulating cell differentiation, proliferation, immune response and blocking apoptosis [2,3]. In mammalian cells, the NFκB/Rel family consists of five members: RelA (p65), RelB, c-Rel, p105/p50 (NFκB1), and p100/p52 (NFκB2). Each family member has a conserved Rel homology domain specifying DNA binding, protein dimerization, and nuclear localization. In most cells, NFκB is composed of a heterodimer of p65 and p50, where the p65 protein is responsible for the transactivation potential. In unstimulated cells, NFκB is sequestered predominantly in the cytoplasm in an inactive complex through interaction with IκB inhibitor proteins. In response to stimulation by a variety of potent activators, such as tumor necrosis factor (TNF)-α, interleukin (IL)-1, phorbol ester (PMA) or lipopolysaccharide [4] and genotoxic agents (doxorubicin, radiation) [5,6], IκBα is rapidly phosphorylated at two conserved NH_2_-terminal serines (Ser-32 and Ser-36) and degraded through a ubiquitin-dependent proteolysis, resulting in the release of NFκB, its translocation into the nucleus and induction of gene transcription. The NFκB has a role in oncogenesis and regulation of cancer therapy sensitivity. Overexpression, amplification, and rearrangements of different genes related to NFκB have been observed in tumors [7]. NFκB is activated in response to various inflammatory stimuli including cytokines, mitogens, bacterial products, viral proteins, and apoptosis-inducing agents [8,9]. Constitutive expression of NFκB leads to activation of several factors involved in cell cycle progression and cell differentiation for cancer metastasis. Inhibition of NFκB activity in tumor cells dramatically reduces cell growth *in vitro *and *in vivo *[10]. NFκB, possibly through the activation of the antiapoptotic genes, plays a key role in the protection of cells against inducers of apoptosis including chemotherapeutic drugs [11]. Several mechanisms including increased expression of NFκB proteins, mutations and/or deletions in IκBα gene, and increased IκBα turnover, are involved in NFκB hyperactivation in tumor cells [7,12]. As such, various therapeutic strategies aim to decrease chronic NFκB hyperactivation by pharmacological as well as phytomedicinal approaches in cancer [13-17]. NFκB-regulated genes are involved in cell death, invasiveness, proliferation, angiogenesis, inflammation and multidrug resistance (MDR). One of the most important mechanisms by which tumor cells resist to cytotoxic effects of a variety of chemotherapeutic drugs (including vinblastine, doxorubicine, etoposide and teniposide, as well as many other cytotoxic agents) is overexpression of the *mdr1 *gene and its product, P-glycoprotein (P-gp) [18].

P-gp is a 180 kDa protein which belongs to the ATP-binding cassette (ABC) superfamily of membrane transporter proteins [19,20]. It is expressed in various tissues, such as kidney tubules, colon, pancreas and adrenal gland, and tumors derived from these tissues are often resistant to chemotherapeutic drugs. Furthermore, *mdr1 *expression is also increased in many relapsing cancers. P-gp is an energy-dependent drug efflux pump that maintains intracellular drug concentrations below cytotoxic levels, thereby decreasing the cytotoxic effects of a variety of chemotherapeutic agents, including anthracyclines, vinca alkaloids, and epipodophyllotoxins [18,21]. P-gp also plays a role in inhibition of drug accumulation and caspase activation in the MDR tumor [22-24]. Of special note, NFκB-mediated drug resistance was found to depend on the regulation of P-gp [25]. In addition, NFκB-dependent regulation of P-gp expression has also been demonstrated in renal tubules or liver [26,27]. By upregulation of P-gp expression, NFκB was found to control drug efflux in cancer cells.

Cancer cells contain multiple signal transduction pathways whose activities are frequently increased due to cell transformation, and these pathways are often activated following cell exposure to established cytotoxic therapies, including ionizing radiation and chemical DNA-damaging agents. Many pathways activated in response to transformation or cytotoxic agents promote cell growth and invasion, which counteract the processes of cell death. As a result of these findings, many drugs with varying specificities have been developed to block the signaling by these cell survival pathways in the hope of killing tumor cells and sensitizing them to toxic therapies [28]. Unfortunately, due to the plasticity of signaling processes within a tumor cell, inhibition of a single growth factor receptor or signaling pathway frequently has only modest long-term effects on cancer cell viability, tumor growth, and patient survival. As a result of this observation, a greater emphasis has begun to be put on multi-target natural compounds, such as polyphenols, withanolides, xanthones, indanones, curcuminoids, which simultaneously inhibit multiple inter-linked signal transduction/survival pathways [14,28-33]. Hopefully, this could limit the ability of tumor cells to adapt and survive, because the activity within multiple parallel survival signaling pathways has been reduced [34]. As such, over the past decades, the efforts of researchers in looking for the new drugs to use in oncology have refocused on natural products [1,34].

One of the expanding directions of modern medicine is based on using of natural phytochemical compounds. Polyphenols or phenolic compounds encompass molecules that possess an aromatic ring bearing one or more hydroxyl substituents. Natural polyphenols can range from simple molecules, such as phenolic acids and flavonoids, to large highly polymerized compounds, such as tannins [35]. This class of phytochemicals can be found in high concentrations in wide varieties of higher plants and their products, such as wine and tea. They were also demonstrated to exert a wide range of biological activities including antioxidant, anticarcinogenic, antiproliferative, antimicrobial anti-inflammatory and apoptosis-inducing activities [36-40].

Various polyphenols have been characterized with respect to their anti-invasive potential. Because invasion is, either directly or via metastasis formation, the main cause of death in cancer patients, development of efficient anti-invasive agents is an important research challenge [31]. Vanden Berghe *et al*. showed that phytoestrogenic soy isoflavones can selectively block nuclear NFκB transactivation of specific NFκB target genes independently of their estrogenic activity in highly metastatic breast cancer cells [16]. In 12-O-tetradecanoylphorbol-13-acetate (TPA)-induced mouse skin tumor, the oligomeric and polymeric polyphenols decreased TPA-induced cell proliferation by attenuating the activation of signaling kinases [c-Jun N-terminal protein kinase (JNK), extracellular signal-regulated protein kinase-1/2 (ERK1/2), p38 protein kinase and Akt], transcription factors [activator protein-1 (AP1) and NFκB] and inflammatory protein [cyclooxygenase-2 (Cox-2)] [41,42]. The NFκB and Akt kinase pathways, which play critical roles in inflammation, vascular homeostasis and angiogenesis, were repressed by the polyphenolic compound deguelin in human vascular endothelial cells, *HT1080 *fibrosarcoma cells and chronic lymphocytic leukemia cells [43-45]. Nitric oxide production was reduced by the green tea polyphenols (-)-Epigallocatechin-3-gallate (EGCG) and black tea theaflavins by suppressing inducible nitric oxide synthase in a breast cancer cell line [46]. The latter treatment blocks nuclear translocation of the transcription factor NFκB as a result of decreased IκB kinase activity. However, anti-cancer effects of polyphenols may also indirectly also involve effects on immune cells at the cancer-inflammation interface. Several studies demonstrated that polyphenolic compounds exhibit anti-inflammatory activity in activated macrophages by inhibiting the NFκB signaling pathway [39,47,48]. Dijsselbloem *et al*. demonstrated that genistein inhibits IL6 gene expression by modulating the transcription factor NFκB in TLR4-stimulated dendritic cells [49]. Pycnogenol inhibits TNFα-induced NFκB activation and adhesion molecule expression in human vascular endothelial cells [50]. Red wine polyphenols, delphinidin and cyanidin inhibit platelet-derived growth factor_AB _(PDGF_AB_)-induced VEGF release in vascular smooth muscle cells by preventing activation of p38 MAPK and JNK [51]. Olive oil polyphenols exert rapid inhibition of p38 and CREB phosphorylation leading to a downstream reduction in COX-2 expression in human colonic adenocarcinoma, Caco-2 cells [52].

Previously, we have already reported the significant anti-cancer activities of quercetin, Siamois 1 and Siamois 2 polyphenols and the withasteroid withaferin A, which hold promise as dietary supplements in nutrition-based intervention in cancer treatment [32,53]. In this study we wanted to further investigate whether interference of Siamois polyphenols and withasteroids with NFκB-dependent apoptosis and inflammatory pathways can sensitize doxorubicin-resistant P-gp-overexpressing K562 erythroleukemic cells for cell death.

## Materials and methods

### Reagents and Chemicals

Quercetin, Kaempferol, and Eriodictyol were from Extrasynthèse (Genay, France), Withaferin A from Chromadex (Irvine, US), whereas home-purified 5,3'-dihydroxy-3,6,7,8,4'-pentamethoxyflavone (WP283) has been described elsewhere [54]. These compounds were stored as 100 mM solutions in DMSO at -20°C. Doxorubicin hydrochloride was kindly provided by Dr. F. Offner (University Hospital UGent). Phorbol-12-myristate-13-acetate (PMA) was purchased from Sigma Chemical Company (St Louis, MO, USA) and stored as 1 mg/ml solution in DMSO at -20°C. Recombinant murine TNF, produced in *Escherichia coli *and purified in our laboratory to at least 99% homogeneity, had a specific biological activity of 8.58 × 10^7 ^IU/ml of protein as determined in a standard TNF cytolysis assay. Reference TNF (code 88/532) was obtained from the National Institute of Biological Standards and Control (Potters Bar, UK). Anti-IκBα, anti-p65 (C20), anti-p50 (NLS), anti-cRel (N), anti-RelB (C19), anti-Fra1(H50), anti-Nrf2 (C10), anti-Bax antibodies were from Santa Cruz Biotechnology (Santa Cruz, CA), anti-p38, anti-p44/42, anti-cfos, anti-cjun, anti-junB, anti-junD from Active Motif, anti-Sirt1 from Biomol, anti-Stat3 from Upstate, anti-histone-H3 antibodies from Abcam and anti-tubulin were from Sigma (Bornem, Belgium). The phospho-specific antibodies directed against p65 Ser536, p38 and p44/42 MAPK, cjun, Akt, MEK were from Cell Signaling (Beverly, CA). Anti-Bcl-2, anti-Bim, anti-Bad, anti-P-Bad antibodies were purchased from Cell Signaling (Boston, MA).

### Cell culture and Cytotoxicity assay

Murine fibrosarcoma L929sA cells were maintained in Dulbecco's modified Eagle's medium supplemented with 5% newborn calf serum, 5% fetal calf serum, 100 units/ml penicillin, and 0.1 mg/ml streptomycin. Twenty-four hours before induction, cells were seeded in multiwell dishes such that they were confluent at the time of the experiment.

Doxorubicin-sensitive erythroleukemic cells (K562) and doxorubicin-resistant erythroleukemic cells (K562/Adr) which overexpress P-gp were grown in RPMI 1640 medium supplemented with 10% fetal calf serum, 100 units/ml penicillin, and 0.1 mg/ml streptomycin, in an incubator at 37°C, 95% humidified, 5% CO_2_. Cultures initiated at a density of 10^5^cells/ml grew exponentially to about 10^6 ^cells/ml in 3 days. K562/Adr cell line was cultured in RPMI 1640 medium in the presence of 100 nM doxorubicin for 72 h, after that the cells were grown in RPMI 1640 medium without doxorubicin for 2 weeks before the experiments. For the assays and in order to have cells in the exponential growth phase, cultures were initiated at 5 × 10^5 ^cells/ml and used 24 h later, reaching a density of about 8-10 × 10^5 ^cells/ml.

The cytotoxicity assay was performed as described previously [55]. Cells (5 × 10^4 ^cells/ml) were incubated in the presence of various concentrations of compounds tested. The viability of cells was determined by MTT reduction. The concentration of compound required for 50% inhibition of the proliferation of cells (IC50) was determined by plotting the percentage of cell growth inhibition (%IC) versus the compound concentration when measured at 72 h. Alternatively, cell cytotoxicity assays were performed by the ToxiLight Assay (Lonza) according to manufacturer's instructions.

### Apoptosis assay

Cells were washed with ice-cold phosphate-buffered saline (PBS) after treatment, and 5 × 10^5 ^cells were stained with annexin V (AnnV)-FITC during 15 min in the dark followed by propidium iodide (PI) staining (human AnnexinV-FITC Detection kit, Bender MedSystems Diagnostics, Vienna, Austria). The stained cells (10^4 ^cells) were measured by flow cytometry (Cytomics FC500 1 laser, Beckman Coulter, Fullerton, USA) and results were expressed as percentage of living (AnnV-, PI-), early apoptotic (AnnV+, PI-), and late apoptotic/dead cells (AnnV+, PI+). The percent of living cells was normalized to 100% living cells incubated in control medium with 0.1% DMSO. All measurements were made in duplicate and averaged.

### IL6 ELISA

IL6 cytokine levels in cell-free culture supernatants were determined using Hu-IL6 Cytoset ELISA kit (Biosource International Inc. Camarillo USA) with detection limits of 15 pg/ml, according to the manufacturer's instructions. Three independent experiments were done, each in triplicate.

### Measurement of caspase-3/7 activity

After appropriate induction, cells were washed with ice-cold PBS and the cytosolic cell lysate was prepared as described previously [56]. Measurement of caspase-3/7 activity was carried out by the incubation of cytosolic cell lysate with fluorogenic substrates, Ac-DEVD-AMC. The release of fluorescent AMC was monitored for 1 h at 37°C at 2-min time intervals in a fluorescence microplate reader (Packard Instrument Co.) using a filter with an excitation wavelength of 360 nm and a filter with an emission wavelength of 460 nm [57,58]. Data are expressed as the increase in fluorescence as a function of time (Δfluorescence/min) normalized with that of cells incubated in control medium with 0.1% DMSO.

### Reporter Gene Analysis

The recombinant plasmid p(IL6κB)_3_50 hu.IL6P-luc+ was described previously [59,60]. Stable transfection of L929sA cells was performed by the calcium phosphate precipitation procedure according to standard protocols [59]. Luciferase and galactosidase reporter assays were carried out according to the manufacturer's instructions (Promega) and have been described previously [59]. Normalization of luciferase activity was performed by measurement of β-galactosidase levels in a chemiluminescent reporter assay Galacto-Light kit (Tropix, Bedford, MA). Light emission was measured in a luminescence microplate reader (Packard Instrument Co.). Luciferase activity, expressed in arbitrary light units, was corrected for the protein concentration in the sample by normalization to the co-expressed β-galactosidase levels. β-Galactosidase protein levels were quantified with a chemiluminescent reporter assay Galacto-Light kit (Tropix).

### Western blot analysis

For the western blot analysis of total cell lysates, cells were washed with ice-cold PBS before lysis in catenine lysis buffer (1% Triton X-100, 1% NP-40, 10 mg/l leupeptine, 10 mg/l aprotinin, 2 mM PMSF, 10 mM sodium fluoride, 10 mM sodium pyrophosphate, 1 mM sodium vanadate). Protein concentration in lysates was measured using BCA™ Protein Assay Kit (Thermo Scientific, Rockford, USA) according to the manufacturer instructions. Lysates were stored at -20°C until assayed.

Before analysis, lysates were diluted to reach equal protein concentration in each sample, and SDS sample buffer was added (0.5 mM Tris-HCl, pH 6.8, 40% glycerol, 9.2% SDS, 5% mercaptoethanol, and 0.05% w/v bromophenol blue), one part of buffer for three parts of diluted lysate. To shear DNA and reduce sample viscosity, samples were heated to 95°C for 5 min, after which they were immediately cooled on ice and microcentrifuged for 5 min. For the western blot analysis of nuclear extract, the nuclear proteins were suspended in SDS sample buffer at the same concentration. The protein samples were separated by 12% SDS-PAGE and electrotransferred onto a nitrocellulose membrane. Blots were probed using the appropriate antibodies and the immunoreactive protein was detected using enhanced chemiluminescence reagents on an Odyssey imaging system (LI-COR Biosciences, Lincoln, USA).

### Electrophoretic Mobility Shift Assay (EMSA)

After treatment, cells were washed with ice-cold PBS and pelleted in 1 ml PBS by centrifugation for 10 min at 2600 rpm (4°C). Preparation of nuclear extracts has been described previously [60]. For EMSA, equal amounts of protein were incubated for 25 min with an NFκB-specific ^32^P-labeled oligonucleotide and binding mix as described previously [32]. For supershift assay, antibodies were preincubated to the sample of interest for 10 minutes prior to incubation with radiolabeled probe [61]. Labeling of the oligonucleotides was performed with [α-^32^P]-dCTP by using Klenow enzyme (Boehringer Mannheim). For EMSA competition assays, 100 fold excess of unlabeled NFκB oligonucleotide was added to the binding mix. The NFκB oligonucleotide comprises the sequence: 5'-AGCTATGT***GGGTTTTCCC***ATGAGC-3', in which the single IL6 promoter-derived NFκB motif is bold and italicized. Samples were loaded on a 6% polyacrylamide gel run in 0.5 × TBE buffer (pH 8) and complexes formed were analyzed using Phosphor Imager Technology.

### RNA isolation and real-time Q-PCR analysis

Total RNA was extracted with the acid guanidinium thiocyanate-phenol-chloroform method using the Trizol reagent (Invitrogen, Merelbeke, Belgium). Reverse transcription was performed on 500 ng of total RNA in a 30 μl total volume. For normalization, cDNA concentrations in each sample were determined prior to quantitative real-time PCR (Q-RT-PCR). The Q-RT-PCR was performed on 5 μl of each condition using Invitrogen Sybr green platinum Supermix-UDG on a iCycler apparatus (Bio-Rad, Eke, Belgium). All amplifications were performed in duplicate or triplicate, and data were analyzedanalyzed using Genex software (Bio-Rad, Eke, Belgium). Data were expressed as mRNA expression normalized with that of cells incubated in control medium with 0.1% DMSO. Q-PCR primers are summarized in Table 1.

**Table 1:** Primers sequences used in the real-time Q-PCR. Note: the abbreviations used in the table: FW indicates forward; REV, reverse.

IL6 FW	GACAGCCACTCACCTCTTCA
IL6 REV	AGTGCCTCTTTGCTGCTTTC
IL8 FW	GCTCTCTTGGCAGCCTTCCTGA
IL8 REV	ACAATAATTTCTGTGTTGGCGC

A/BFL-1 FW	GATTTCATATTTTGTTGCGGAGTTC
A/BFL-1 REV	TTTCTGGTCAACAGTATTGCTTCAG

MCP1 FW	ACTCTCGCCTCCAGCATG
MCP1 REV	TTGATTGCATCTGGCTGAGC

A20 FW	CCTTGCTTTGAGTCAGGCTGT
A20 REV	TAAGGAGAAGCACGAAACATCGA

CYCLIND1 FW	CGCCCCACCCCTCCAG
CYCLIND1 REV	CCGCCCAGACCCTCAGACT

VEGF FW	GCCTCCCTCAGGGTTTCG
VEGF REV	GCGGCAGCGTGGTTTC

MDR1 FW	CTGCTTGATGGCAAAGAAATAAAG
MDR1 REV	GGCTGTTGTCTCCATAGGCAAT

## Results

### Siamois polyphenols and the withasteroid withaferin A dose dependently inhibit NFκB-driven reporter gene expression

**Figure 1:**
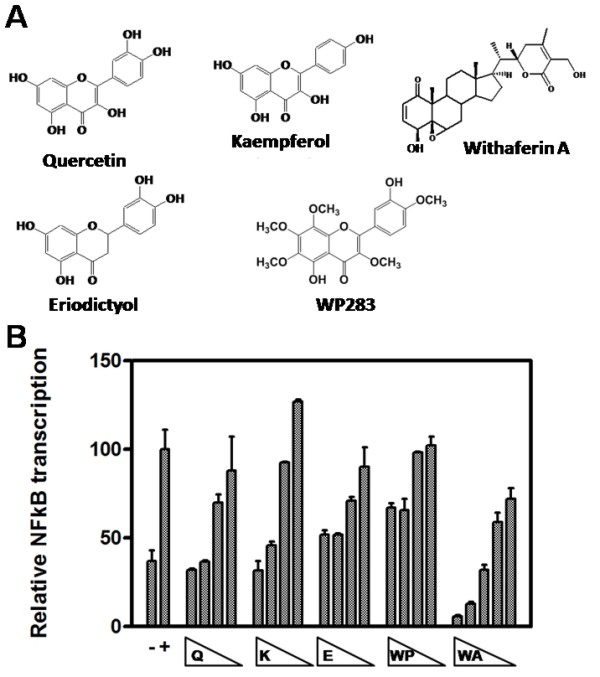
**Siamois polyphenols and withasteroids dose-dependently inhibit NFκB-dependent reporter gene expression**. **A) **Chemical formulas of quercetin, kaempferol, eriodictyol, WP283, and withaferin A. **B) **L929sA cells, stably transfected with p(IL6κB)_3_50 hu.IL6P-luc+, were left untreated or were pretreated with a concentration range of 100-50-25-12.5 μM Siamois polyphenol (quercetin, kaempferol, eriodictyol, WP283) or 3-1.5-0.75-0.38 μM withaferin A for 2 h, and then stimulated with 2000 IU/ml TNF for 4 h. NFκB-dependent reporter gene expression was normalized for PGK housekeeping reporter gene expression. Data of two independent experiments, each done in triplicate, are presented as mean ± S.E.M.

As anti-cancer properties of various polyphenols have been linked to inhibition of the inflammatory transcription factor NFκB, we first compared potential anti-inflammatory properties of the Siamois polyphenols quercetin, kaempferol, eriodictyol, WP283 and the withasteroid withaferin A (Fig. 1A) in NFκB-driven reporter gene assays. First, we performed a dose response experiment on L929sA cells, stably transfected with a TNF-inducible NFκB-driven reporter gene construct with a minimal IL6 promoter (p(IL6κB)_3_-50 hu.IL6P-luc+) and a constitutively expressed reporter gene construct (pPGκBGeobpA) controlled by the phosphoglycerokinase promoter [60] for normalization of reporter gene expression. Upon TNF treatment, significant promoter induction can be observed with the NFκB-driven reporter gene construct, which can be reversed with quercetin, kaempferol, eriodictyol, WP283 or withaferin A in a dose-dependent manner. IC50 values for NFκB inhibition for the different Siamois polyphenols vary in the concentration range of 30 to 50 μM and 0.5-1 μM for withaferin A (Fig. 1B).

### Siamois polyphenols and withaferin A inhibit endogenous NFκB target gene transcription in K562 and K562/Adr cells, irrespective of doxorubicin sensitivity

**Figure 2:**
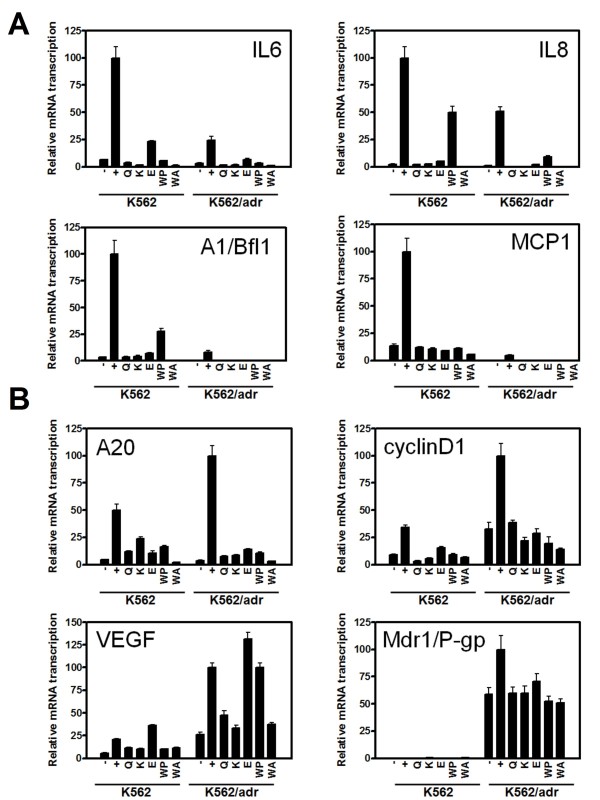
**Siamois polyphenols and withasteroids inhibit endogenous NFκB-dependent transcription in K562 and K562/Adr cells**. K562 and K562/Adr cells were pretreated with 100 μM of quercetin, kaempferol, eriodictyol, WP283, or 6 μM of withaferin A for 2 h followed by incubation with PMA (0.1 μg/ml) for 3 h. Total RNA was isolated and mRNA was converted into cDNA. Relative mRNA levels were quantified by QPCR by specific primer sets for **A) **IL6, IL8, A1/Bfl1, MCP1, **B) **A20, cyclin D1, VEGF, mdr1. Specific mRNA transcription levels were normalized by transcription levels of cells incubated in control medium with 0.1% DMSO. Data of two independent experiments, each done in triplicate, are presented as mean ± S.E.M.

To validate our reporter gene expression results in more specific cancer settings, we further studied Siamois polyphenol effects in K562 and K562/Adr cells, which may demonstrate different NFκB activation status related to doxorubicin sensitivity [62]. Since NFκB hyperactivation is involved in chemoresistance, we next evaluated whether different types of NFκB inhibitors may have different effects on endogenous NFκB target genes in K562 and K562/Adr cells, involved in inflammation, metastasis (IL6, IL8, MCP1, A20), cell cycle (cyclin D1), angiogenesis (VEGF), multidrug resistance (mdr1/P-gp), and apoptosis (A1/Bfl1). Cells were pretreated with Siamois polyphenols or withaferin A for 2 h, either or not following 3 h treatment of PMA, after which RNA was isolated and mRNA levels of interest were quantified by Q-PCR with specific primers. As illustrated in Fig. 2, NFκB target genes are potently induced by PMA in both cell types. Surprisingly, NFκB target genes are differentially expressed in K562 as compared to K562/Adr cells. More particularly, whereas IL6, IL8, MCP1 and A1/Bfl1 reveal stronger transcription in K562 cells, A20, cyclin D1, VEGF and P-gp, are preferentially expressed in K562/Adr cells. Furthermore, repression of PMA-inducible NFκB target genes can be observed in K562 and K562/Adr cells, irrespective of levels of Mdr1/P-gp expression. Interestingly, although NFκB inhibitors can completely reverse the effect of PMA on P-gp expression in K562/Adr cells, its basal transcription levels cannot be further reversed to the background P-gp levels as observed in K562 cells.

Finally, efficacy of target gene repression seems also to be compound- and target gene-specific. Altogether, these results demonstrate differential inhibitory effects of Siamois polyphenols and withasteroids on target genes involved in inflammation, metastasis, cell cycle, angiogenesis, multidrug resistance, and anti-apoptosis in doxorubicin-sensitive or -resistant K562 cells.

### Siamois polyphenols and withaferin A inhibit endogenous IL6 protein expression in K562 and K562/Adr cells, irrespective of doxorubicin sensitivity

**Figure 3:**
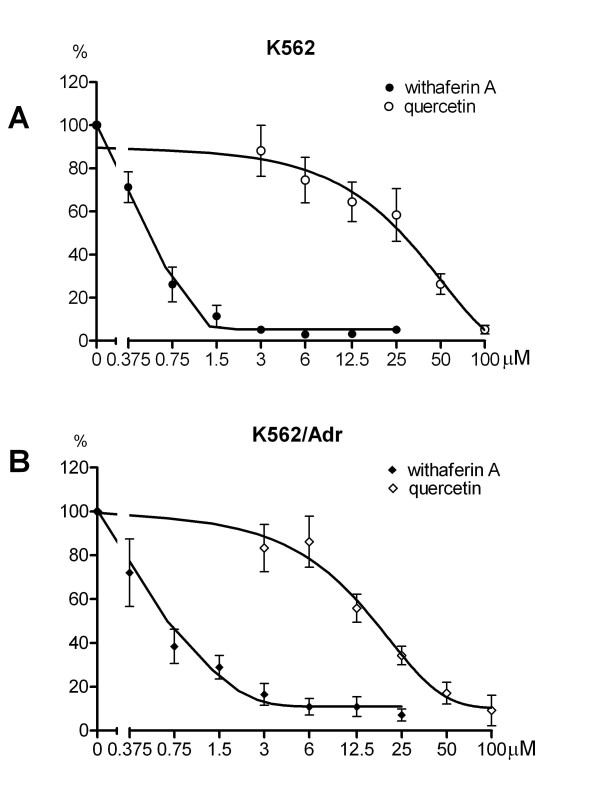
**Inhibitory effect of withaferin A and quercetin on IL-6 protein production in K562 and K562/Adr cells**. K562 and K562/Adr cells were pretreated with quercetin or withaferin A in different concentrations (as indicated in Figure). for 3 h, followed by incubation with PMA (100 ng/ml) for 15 h. Cell-free medium supernatant was collected for ELISA quantification of secreted levels of IL-6 protein. Data are presented as percent of control (zero concentration of withaferin A or quercetin), as mean ± S.E.M. of three independent experiments, each done in triplicate. Following IC50 values were obtained: 0.54 μM withaferin A and 29.5 μM quercetin for K562 cells; 0.58 μM withaferin A and 13.2 μM quercetin for K562/Adr cells.

To evaluate whether inhibition of endogenous NFκB target genes is also translated at the protein level, we performed IL6 ELISA of IL6 protein secreted into the medium of K562 and K562/Adr cells, pretreated with different doses of quercetin or withaferin A for 3 h, either or not following 15 h treatment of PMA, after which medium was collected to determine IL6 protein levels. As illustrated in Fig. 3, a comparable dose dependent decrease in IL6 protein levels can be observed in both cell types. In line with the NFκB reporter gene results, inhibition of IL6 protein expression can be achieved with lower concentrations withaferin A than quercetin.

### All the Siamois polyphenols and withaferin A prevent IκB degradation but the compounds selectively interfere with p38, ERK MAPK, MEK1 and Akt kinase activation

**Figure 4:**
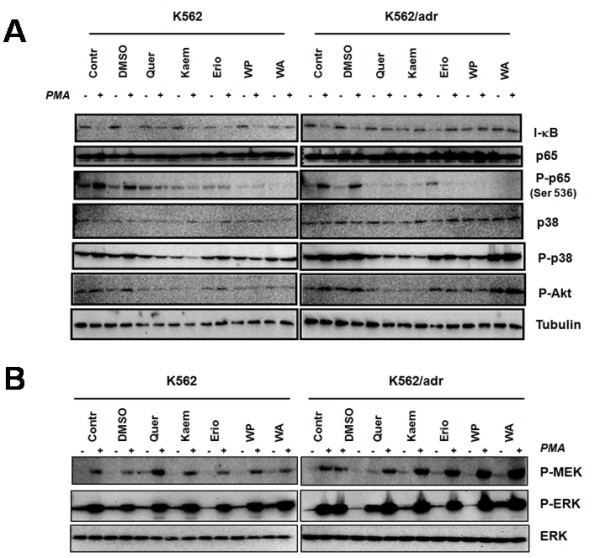
**Selective effects of Siamois polyphenols and withasteroids on the NFκB signaling pathway**. K562 and K562/Adr cells were either or not pretreated with 100 μM of quercetin, kaempferol, eriodictyol, WP283 or 6 μM of withaferin A for 2 h followed by incubation with PMA (0.1 μg/ml) for 30 minutes. Total cell lysates were prepared in SDS-Laemmli sample buffer and extracts were analyzed for protein expression levels of **A) **IκB, p65, P-Ser536 p65, P-p38, P-Akt, tubulin, **B) **P-MEK1, P-ERK, ERK, respectively.

As NFκB target gene expression encompasses multiple regulatory steps, including IκB degradation, NFκB translocation, NFκB/DNA binding and NFκB transactivation, we next aimed to dissect which regulatory steps are affected by Siamois polyphenols in K562 and K562/Adr cells. Since IκBα degradation is required for liberation and subsequent translocation of NFκB to the nucleus, we determined Siamois polyphenol effects on PMA-induced IκBα protein degradation in K562 and K562/Adr cells. As maximal degradation of IκBα is observed between 15-30 minutes after PMA treatment (data not shown), we next measured effects of Siamois polyphenols and withaferin A on IκB degradation following 2 h pretreatment and 30 minutes cotreatment with PMA. From Fig. 4A, it can be observed that all tested compounds reduce IκB degradation in both cell types. Along the same line, all tested compounds significantly reduce basal and/or PMA-inducible p65 Ser536 phosphorylation in both cell types. Altogether, these results suggest that activation of NFκB and subsequent translocation of NFκB for gene induction is significantly reduced in presence of Siamois polyphenols and the withasteroid withaferin A.

As target gene-specific effects are also depending on p65 phosphorylation status and epigenetic settings, dynamically controlled by multiple kinase pathways, i.e. Akt, MAPK, MSK, PKA, we next measured P-Akt, P-p38, P-ERK levels in the various experimental conditions in both cell types. A significant reduction of basal and PMA-induced P-Akt and P-p38 levels can be observed upon treatment with quercetin and kaempferol, but not with withaferin A in both K562 cell types (Fig. 4A), whereas P-ERK levels do not reveal significant inhibition (Fig. 4B). In contrast weak ERK stimulation could rather be observed with withaferin A and quercetin (Fig. 4B). Western analysis against p38 and ERK protein levels confirms equal protein loading in the various experimental setups (Fig. 4A). Interestingly, Siamois polyphenols and withaferin A demonstrate increased MEK1-phosphorylation in K562/Adr cells, suggesting that uptake of compounds is not impaired in P-gp-overexpressing K562/Adr cells.

Altogether, besides significant inhibition of IκB degradation and NFκB p65 Ser536 phosphorylation by Siamois polyphenols and withaferin A, compound-specific regulation of p38, ERK, Akt and MEK kinases could be observed, which may further interfere with nuclear transcriptional regulation of NFκB target genes [63-65].

### K562 and K562/Adr cells reveal distinct nuclear regulation of NFκB, AP1, Nrf2 and Sirt1 proteins

**Figure 5:**
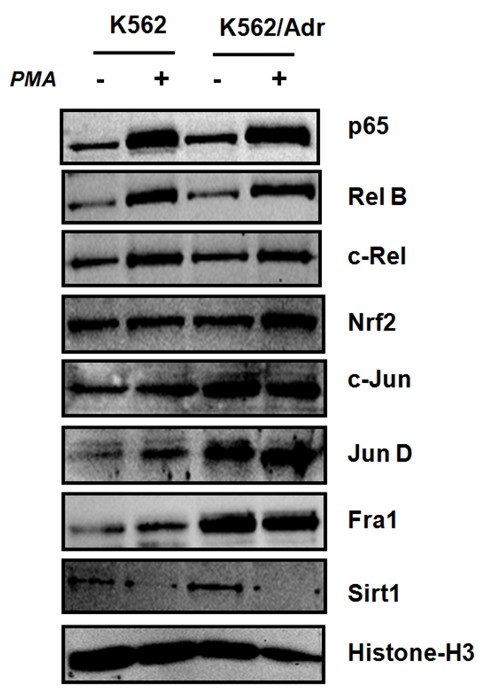
**K562 and K562/Adr cells reveal different nuclear regulation of NFκB, AP1, Nrf2 transcription factors and Sirt1 cofactors**. K562 and K562/Adr cells were treated with PMA (0.1 μg/ml) for 30 minutes. Nuclear cell lysates were prepared in SDS-Laemmli sample buffer and extracts were analyzed for protein expression levels by Western analysis of NFκB p65, RelB, cRel, Nrf2, AP1 cjun, junD, Fra1, Sirt1, respectively. Comparable protein loading was verified with Histone H3 antibodies.

As K562 and K562/Adr demonstrate differential regulation of NFκB target genes, we next explored whether both cell types may show different nuclear regulation of potential cooperative transcription factors (i.e. AP1, Nrf2) or cofactors (Sirt1) which might coregulate NFκB target genes. As can be observed from Fig. 5, basal levels of nuclear NFκB p65, AP1 c-Jun, JunD and Fra1 are significantly increased in K562/Adr cells, but not of cRel and RelB. This confirms previous observations on doxorubicin-resistant MCF7 cells, in which AP1 transcription factors were demonstrated to be responsible for upregulation of P-gp/Mdr1 [66]. Furthermore, PMA treatment significantly increases nuclear levels of NFκB p65, RelB, c-Rel. Of special note, increased nuclear levels of Nrf2 upon PMA treatment are more pronounced in K562/Adr than in K562 cells. Only recently, involvement of Nrf2 has been demonstrated in chemoresistance [67]. Also in line with previous studies on the role of Sirt1 in chemoresistance, basal Sirt1 levels are slightly increased in doxorubicin-resistant K562/Adr cells. More particularly, Sirt1 was found to positively contribute in P-gp/Mdr1 expression [68]. Altogether, our results demonstrate that activities of NFκB p65, AP1 cjun, junD, Fra1, Nrf2 transcription factors and Sirt1 cofactors are increased in doxorubicin-resistant K562/Adr cells.

### NFκB, AP1 DNA-binding profiles in K562 and K562/Adr cells show qualitative and quantitative differences

**Figure 6:**
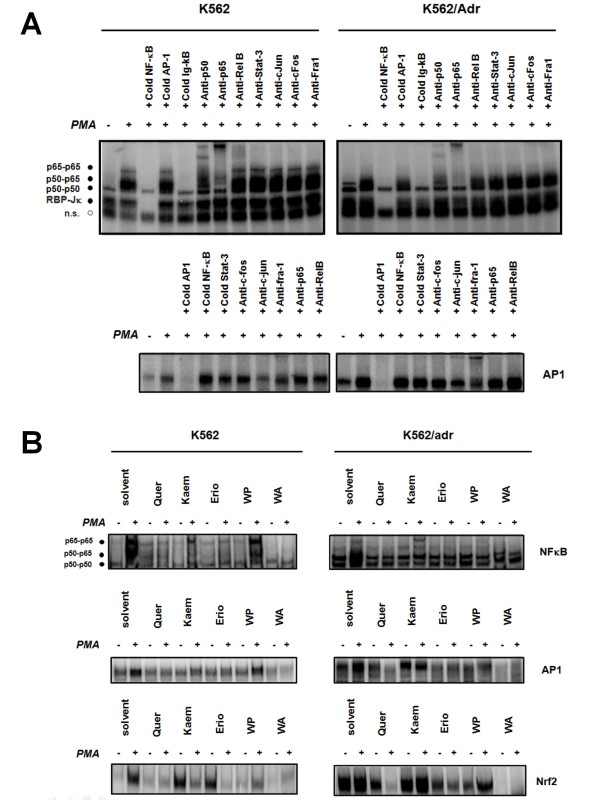
**K562 and K562/Adr cells show qualitative and quantitative differences in NFκB and AP1 DNA binding profiles**. **A) **K562 and K562/Adr cells were pretreated with PMA (0.1 μg/ml) for 30 minutes. Nuclear lysates were analyzed for NFκB/DNA and AP1/DNA binding with a radiolabeled IL6 κB site- or AP1 motif-containing probe. Binding complexes formed were analyzed by EMSA. Loading of equal amounts of protein was verified by comparison with the binding activity of the repressor molecule recombination signal sequence-binding protein Jκ (RBP-Jκ). Specificity of the various complexes bound is demonstrated by supershift analysis with NFκB- and AP1-specific antibodies, as well as by competition with 100-fold excess cold oligonucleotide. **B) **K562 and K562/Adr cells were pretreated with 100 μM of quercetin, kaempferol, eriodictyol, WP283, or 6 μM of withaferin A for 2 h followed by incubation with PMA (0.1 μg/ml) for 30 minutes. Cell lysates were fractionated for cytoplasmic and nuclear extracts which were analyzed for NFκB, AP1, or Nrf2-dependent DNA binding with specific radiolabeled probes. Binding complexes formed were analyzed by EMSA.

To compare DNA-binding properties of NFκB and AP1 in K562 and K562/Adr cells, we performed electrophoretic gel shift mobility assays (EMSA) and supershift analysis in response to PMA stimulation. Fig. 6A reveals that both cell types show inducible NFκB/DNA binding, whereas basal NFκB/DNA binding is slightly elevated in doxorubicin-resistant K562/Adr cells, in line with observations that doxorubicin can elevate basal NFκB activation via DNA damage pathways [69]. Also, K562 and K562/Adr cells show different composition of NFκB/DNA binding complexes. Interestingly, despite increased levels of NFκB/DNA binding observed in K562/Adr cells, it has been demonstrated that NFκB phosphorylation/acetylation levels are reduced, which affects its transcriptional properties for specific subsets of NFκB target genes [70,71]. Along the same line, supershift analysis reveals subtle differences in the heterodimer/homodimer composition of DNA-bound NFκB and AP1-binding complexes in both cell types. Supershift analysis reveals at least three different NFκB/DNA-binding complexes including p65-p65, p50-p65, and p50-p50. In K562/Adr cells, basal NFκB/DNA binding of the p50-p65 complex appears to be increased relative to K562 cells. Similarly, increased basal and inducible AP1 binding is detected in K562/Adr cells in comparison with K562 cells, in line with increased levels of nuclear AP1 members. Furthermore, although both cell types demonstrate PMA inducible NFκB/DNA binding, K562 cells show higher intensity of p65-p65 heterodimers but comparable amounts of p50-p65 and p50-p50 DNA-binding complexes in comparison to K562/Adr cells (Fig. 6A). Concerning AP1-binding complexes, increased Fra1 levels can be detected in K562/Adr cells as compared to K562 cells. EMSA competition with excess of unlabeled NFκB or AP1 DNA-binding motifs further demonstrates specificity of the DNA-bound NFκB, RBP-Jκ and AP1-binding complexes.

### Siamois polyphenols quercetin, eriodictyol and withaferin A strongly inhibit DNA binding of NFκB, AP1 and Nrf2

To verify whether transcriptional repression of target genes involved in inflammation, anti-apoptosis, angiogenesis, metastasis, drug resistance by Siamois polyphenols and withaferin A could be the consequence of inhibition of NFκB, AP1 or Nrf2 TF/DNA binding in K562 and K562/Adr cells, we performed EMSA experiments with nuclear extracts from cells treated with PMA alone, or following pretreatment with Siamois polyphenols. As shown in Fig. 6B, basal constitutive p50-p50 and p50-p65 NFκB/DNA-binding activity in K562/Adr is increased as compared to K562 cells. PMA stimulation again increases p50-p50 and p50-p65 NFκB/DNA binding in both cell types whereas p65-p65 homodimers demonstrate stronger DNA binding in K562 only. Furthermore, treatment with different Siamois polyphenols and withaferin A causes strong to moderate inhibition of the basal and inducible p50/p65 NFκB/- and AP1/DNA-binding complexes, as shown in Fig. 6B[61]. Along the same line, Nrf2/DNA binding is increased in K562/Adr cells as compared to K562 cells, whereas Siamois polyphenols and withaferin A are able to reduce basal and PMA-inducible Nrf2 binding in both cell types [67,72]. Among the different Siamois polyphenols tested, quercetin and eriodictyol show the strongest inhibition of TF/DNA binding, whereas kaempferol and WP283 are less effective. Nevertheless, transcriptional inhibition of the various target genes (Fig. 6B) by Siamois polyphenols and withaferin A is regulated at multiple levels and depends on DNA-binding properties of NFκB, AP1, Nrf2 transcription factors, nuclear cofactor dynamics, as well as epigenetic settings [64,71,73,74]. Of special note, although Siamois polyphenols and withaferin A are able to reverse inducible NFκB/DNA binding in K562/Adr cells, constitutive NFκB/DNA-binding levels cannot be further decreased to levels observed in K562 cells.

### Siamois polyphenols and withaferin A reduce cell viability in both K562 and K562/Adr cells

**Figure 7:**
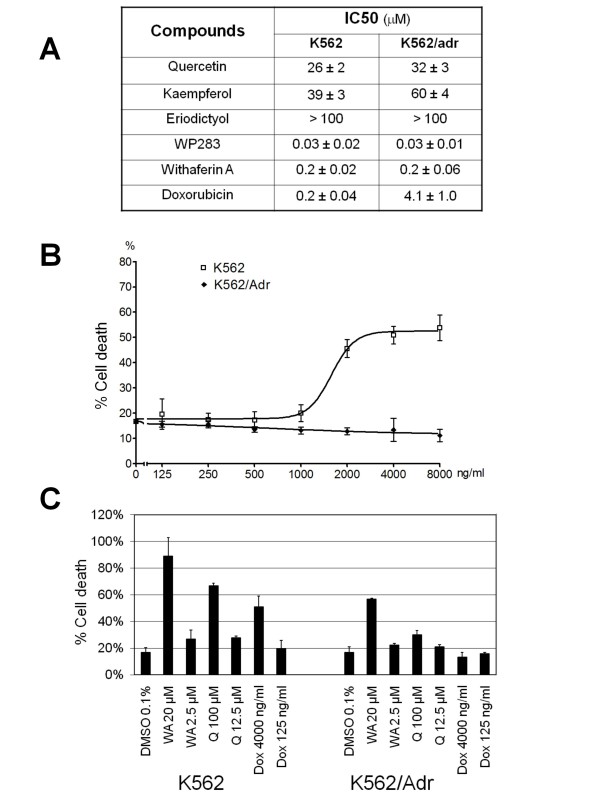
**Cytotoxic effects of Siamois polyphenols and withaferin A in K562 and K562/Adr cells**. **A) **K562 and K562/Adr cells were treated with various concentrations of quercetin, kaempferol, eriodictyol, WP283 or withaferin A for 72 h. Cell survival was determined by mitochondrial MTT assay and IC50 values were determined for cytotoxicity of the different compounds. **B**) K562 and K562/Adr cells were treated with various concentrations doxorubicin for 48 h. % Cell cytotoxicity was determined by ToxiLight assay. **C**) K562 and K562/Adr cells were treated with various concentrations withaferin A or quercetin for 48 h. % Cell cytotoxicity was determined by ToxiLight assay.

K562 and K562/Adr cells which are sensitive or resistant to doxorubicin, respectively, were incubated with doxorubicin, withaferin A or Siamois polyphenols, including quercetin, kaempferol, eriodictyol and WP283 to evaluate cytostatic and/or cytotoxic activity of the various compounds. After 72 h, cell survival was determined by the MTT cell viability assay and the IC50 values are summarized in Fig. 7A. Among Siamois polyphenols, WP283 and eriodictyol exhibit the strongest and weakest effects in mitochondrial reduction of tetrazolium salts to formazan. Of particular interest, K562 and K562/Adr cells reveal comparable sensitivity to Siamois polyphenols and withaferin A, whereas IC50 values for doxorubicin show a 20-fold higher sensitivity in sensitive K562 cells, as compared to resistant K562/Adr cells. These results indicate a pronounced cellular resistance for doxorubicin as compared to Siamois polyphenols and withaferin A.

To exclude any potential artefacts that may come from interaction of intracellular polyphenols with MTT, which could be directly reduced by these compounds [75], we have also measured cytotoxic effects of quercetin, withaferin A and doxorubicin with a bioluminescent luciferase/luciferin ATP based cytotoxicity assay (ToxiLight). In accordance with MTT results, K562/Adr cells show cellular resistance to doxorubicin (Fig. 7B). Furthermore, K562 cells show high sensitivity to both withaferin A and quercetin, while K562/Adr cells show significantly reduced sensitivity to quercetin, and their sensitivity to withaferin A is only partially lost in comparison to K562 cells (Fig. 7C).

### Withaferin A, but not Siamois polyphenols, induces execution of apoptosis

**Figure 8:**
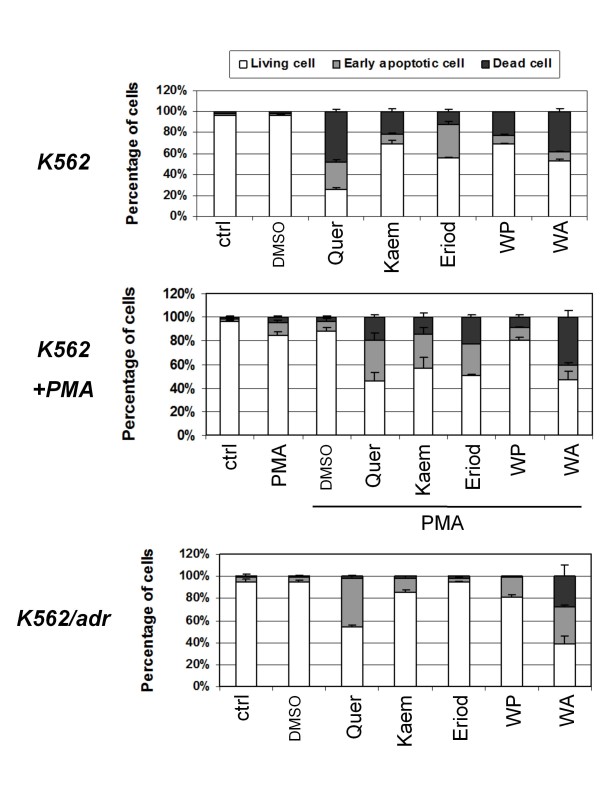
**Apoptotic effects of Siamois polyphenols and withasteroids in K562 and K562/Adr cells**. Determination of early (AnnexV+, PI-) and late (AnnexV+, PI+) apoptotic cells by flow cytometric evaluation (AnnexinV-FITC and PI staining) of mean normalized percentages of living K562 and K562/Adr cells, either left untreated or treated with 100 μM of quercetin, kaempferol, eriodictyol, WP283 or 6 μM of withaferin A, or cotreated with PMA (0.1 μg/ml) for 48 h. Data of two independent experiments, each done in duplicate, are presented as mean ± S.E.M.

Next, K562 and K562/Adr cells were incubated for 48 h with Siamois polyphenols or withaferin A, followed by annexin V-FITC/PI double staining and FACS analysis to quantify early annexin V-FITC positive) and late (annexin V-FITC/PI double positive) apoptotic cells. The relative percentage of apoptotic/living cells in the different experimental setups in K562 and K562/Adr cells, following 48 h treatment are represented as a bar graph in Fig. 8. Interestingly, although both cell types show comparable early apoptotic cell populations in presence of the different Siamois polyphenols, late apoptotic cells only accumulate in K562 cells. In contrast to Siamois polyphenols, only withaferin A is able to trigger late apoptosis in K562/Adr cells. Furthermore, although the concentrations applied of the different Siamois polyphenols closely relate to the IC50 values determined in MTT assay (Fig. 8), FACS analysis (Fig. 8) reveals significant variation in apoptosis efficacy between the different polyphenol compounds. The latter suggests significant discrepancies between MTT cell viability assays revealed by mitochondrial reduction of tetrazolium salts and cell survival score measured by Annexin V/PI apoptosis FACS assay [75]. Indeed, it is of utmost importance to perform multiple, methodologically unrelated assays to quantify dying and dead cells [75,76].

Next, as apoptotic threshold in compound-treated K562/Adr cells may be higher due to elevated basal anti-apoptotic activity of NFκB, AP1 and Nrf2, we wanted to further evaluate whether increasing activity of NFκB, AP1 and Nrf2 by PMA treatment in K562 cells could similarly protect compound-treated K562 cells from late apoptosis in analogy to K562/Adr cells. However, although the relative number of late apoptotic cells decreases upon cotreatment of K562 cells with PMA and Siamois polyphenol inhibitors (Fig. 8), execution of apoptosis is not completely blocked because Siamois polyphenols are able to partially counteract PMA effects on NFκB, AP1 and Nrf2. Along the same line, Siamois polyphenols cannot overcome the late apoptosis block in K562/Adr cells, despite efficient inhibition of NFκB, AP1 and Nrf2. This suggests that execution of apoptosis in K562/Adr cells is only in part determined by transcriptional activity of NFκB, AP1 and Nrf2. Remarkably, although withaferin A, and quercetin both dose dependently inhibit NFκB, AP1 and Nrf2 in K562/Adr cells, only withaferin A is able to trigger late apoptosis and overcome the apoptosis block in K562/Adr cells, indicating that withaferin A may also affect other death-inducing pathways/mechanisms.

### Withaferin A and quercetin induce early and late caspase activation respectively

**Figure 9:**
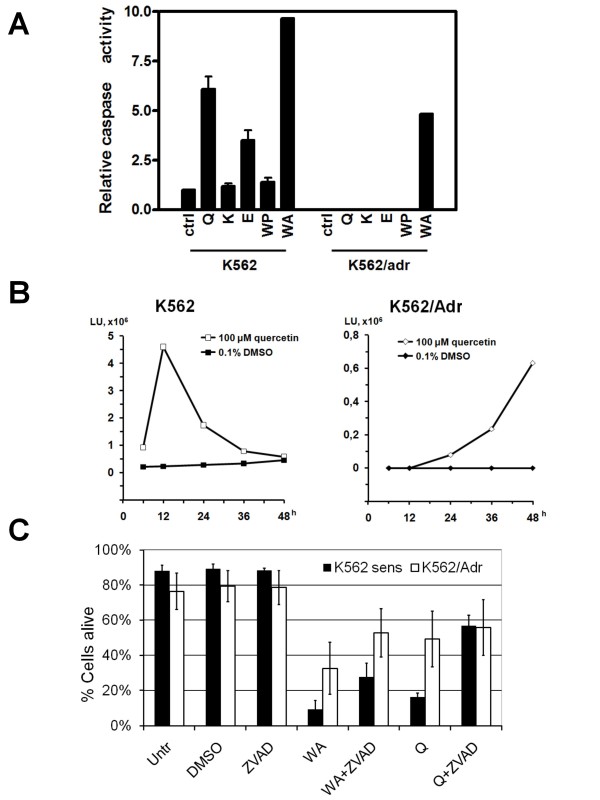
**Caspase 3/7 activation by Siamois polyphenols and withaferin A in K562 and K562/Adr cells**. **A) **K562 and K562/Adr cells were either left untreated or treated with 100 μM of quercetin (Q), kaempferol (K), eriodictyol (E), WP283 (WP) or 6 μM of withaferin A (WA) for 12 h. Caspase-3 activity present in cell lysates was determined by a fluorescent *in vitro *assay upon incubation of cell lysate with Ac-DEVD-fmk substrate. **B) **K562 and K562/Adr cells were either left untreated or treated with 100 μM quercetin for 6, 12, 24, 36 or 48 h. Caspase-3 activity present in cell lysates was determined by a fluorescent *in vitro *assay upon incubation of cell lysate with Ac-DEVD-fmk substrate. **C) **Percentage of living cells, following treatment with withaferin A (10 μM) or quercetin (100 μM) in the presence or absence of ZVAD fmk (50 μM), inhibitor of caspases. In all conditions, except control, cells were cultivated in medium containing 1% DMSO. After 48 h of cultivation, % dead cells were stained by annexin V/propidiumiodide followed by flow cytometry measurement. % Living cells is obtained by subtracting % dead cells. Data of two independent experiments, each done in duplicate, are presented as mean ± S.E.M.

In addition to propidium iodide as a late apoptotic FACS marker, we next measured biochemical activation of the executioner caspases-3/7 in K562 and K562/Adr cells exposed to PMA, Siamois polyphenols and/or withaferin A in a fluorescent caspase substrate assay. In this respect, K562 and K562/Adr cells were treated for 12 h with PMA, Siamois polyphenols and/or withaferin A, after which caspase activity present in the cell lysates was measured in presence of the caspase substrate Ac-DEVD-fmk, which elicits fluorescence upon its cleavage. From Fig. 9A it can be noticed that Siamois polyphenols increase caspase-3/7 activity only in K562, but not in K562/Adr cells, which is in good accordance with lack of late apoptosis observed in K562/Adr cells. In contrast to Siamois polyphenols, withaferin A is able to trigger caspase-3/7 activity in both cell types Fig. 9A. Interestingly, upon evaluation of quercetin-dependent activation of caspase-3/7 at later time points, i.e. 36 h and 48 h, we observed a delayed but significant increase in caspase-3/7 activity, which could be responsible for attenuation of late apoptosis events in K562/Adr cells exposed to quercetin (Fig. 9B). Kinetic differences in apoptosis by withaferin A and quercetin will be further discussed in paragraphs below. Further support for involvement of caspases in withaferin A- and quercetin-dependent cell death in K562 and K562/Adr cells follows from experiments in presence of the pan-caspase inhibitor ZVAD-fmk. Briefly, K562 and K562/Adr cells were grown for 48 h in withaferin or quercetin in presence or absence of ZVAD-fmk. As can be observed from Fig. 9C, withaferin A and quercetin both trigger cell death in K562 cells which can partially be reversed with the pan-caspase inhibitor ZVAD-fmk. Also in K562/Adr cells, withaferin-dependent apoptosis effects can be partially reversed with ZVAD-fmk, whereas ZVAD-fmk effects on the quercetin-dependent apoptosis setup are much weaker, since quercetin induced caspase-3/7 activation is less efficient or slower than for withaferin treatment.

### PARP cleavage by withaferin A in K562 and K562/Adr cells is reversible by thiol donors

**Figure 10:**
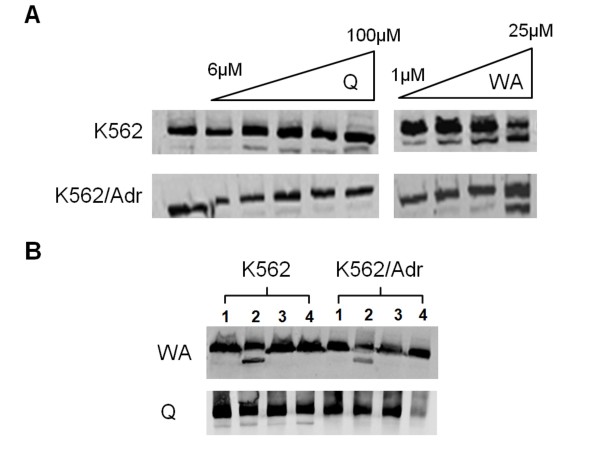
**Differential efficacy of PARP cleavage by quercetin and withaferin A in K562 and K562/Adr cells**. **A) **K562 and K562/Adr cells were either left untreated or treated with different doses of quercetin or withaferin A for 24 h. Cell lysates were analyzed for PARP cleavage by Western analysis. **B) **K562 and K562/Adr cells were either left untreated (1), 24 h treated with 100 μM of quercetin or 10 μM withaferin A (2), 24 h treated with 100 μM quercetin or 10 μM withaferin A following 2 h prior WA preincubation with 1 mM DTT (3), or 24 h treatment with 1 mM DTT (4). Corresponding cell lysates were analyzed for PARP cleavage by Western analysis.

Next, we further investigated by Western analysis whether caspase activation results in cleavage of PARP, caspase substrate and standard marker for apoptosis (Fig. 10). K562 and K562/Adr cells were incubated for 24 h with different doses of withaferin A or quercetin. In line with our FACS data and toxicity assays, high doses of withaferin A trigger significant PARP cleavage in K562 cells and to a lesser extent in K562/Adr cells (Fig. 10A). Also quercetin triggers PARP cleavage in K562 cells, although in K562/Adr cells PARP cleavage is strongly impaired or delayed. Since we and others previously demonstrated reversal of biological effects of withaferin in presence of excess amounts of thiol donors (i.e. mercaptoethanol or dithiothreitol (DTT)) [32,77,78], we have further tested whether PARP cleavage by withaferin A could also be prevented in presence of DTT. Interestingly, PARP cleavage by withaferin A in K562 and K562/Adr cells was completely blocked following prior incubation with DTT, illustrating a major role for thioalkylation targets in withaferin A-dependent cytotoxicity (Fig. 10B). In contrast, quercetin effects on PARP cleavage could not be attenuated by DTT in K562 cells.

### Effect of withaferin A and quercetin on apoptosis-related proteins in K562 and K562/Adr cells

**Figure 11:**
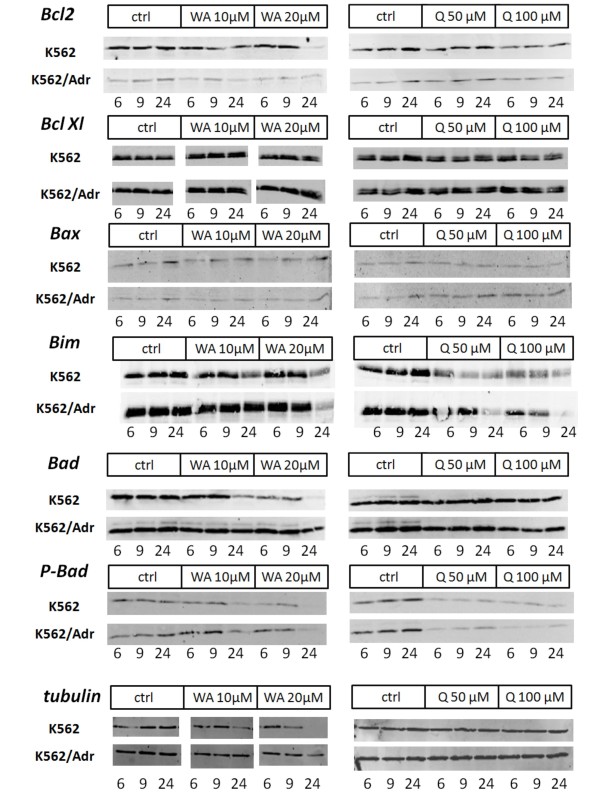
**Effects of withaferin A and quercetin on intracellular apoptosis-related proteins in K562 and K562/Adr cells**. K562 and K562/Adr cells were either left untreated or treated with different doses of quercetin or withaferin A for 6, 9 or 24 h. Equal protein amounts of corresponding cell lysates were analyzed for protein levels of Bcl2, Bcl_XL_, Bax, Bim, Bad, P-Bad and tubulin by Western analysis.

The Bcl2 family of antiapoptotic proteins (Bcl2, Bcl_XL_), proapoptotic families of BH123 (Bax) and BH3 (Bim) proteins represent 3 major classes of intracellular regulators of apoptosis. As such, we performed Western analysis to evaluate effects of withaferin A and quercetin on Bcl2, Bcl_XL_, Bax and Bim protein levels in K562 and K562/Adr cells, exposed for different time periods to high or low concentrations of the compounds. In Fig. 11 we demonstrate that in K562 cells, withaferin A and quercetin time-dependently and dose-dependently decrease the levels of Bcl2, Bim and P-Bad protein, whereas Bcl_XL _and Bax levels remain largely unaffected in any condition. Similar results were obtained in K562/Adr cells, although decrease of protein levels is generally delayed (starts to appear at 24 h rather than 9 h). Furthermore, withaferin A decreases protein levels of Bad whereas quercetin has no effect. Finally and of special interest, in analogy to various anti-cancer drugs acting on the cytoskeleton and interfering with tubulin dynamics, withaferin A seems to significantly decrease tubulin protein levels, whereas no effect can be observed in presence of quercetin.

## Discussion

Extensive studies indicate that both hyperactivation of NFκB and overexpression of multidrug transporters play important roles in cancer chemoresistance [7,18,25,27,62,68]. Since expression of the multidrug transporter P-gp was found to be NFκB-dependent, it is believed that NFκB inhibitors can decrease P-gp expression and restore chemosensitivity [1,14]. However, our studies have shown that the picture is more complicated. Previously, we have already demonstrated apoptosis of MDA-MB435 cells in presence of Siamois polyphenols in a xenograft model in vivo [53]. Furthermore, the NFκB inhibitor withaferin A has been described as a promising drug for cancer chemotherapy and radiosensitization [32,79,80]. Now, we further analyzed whether withaferin A or Siamois polyphenols quercetin, kaempferol, eriodictyiol, and WP283 hold therapeutic promise as NFκB-inhibitors for chemosensitization of doxorubicin resistant K562/Adr erythromyelogenous leukemia cells. In NFκB reporter gene studies, we compared dose-dependent repression of luciferase gene expression in response to Siamois polyphenols quercetin, kaempferol, eriodictyiol, and WP283 with IC50 values in the range of 0.1-50 μM respectively. Furthermore, upon comparing endogenous gene transcription and protein expression of specific NFκB target genes, we observed comparable potencies in NFκB-dependent gene repression by Siamois polyphenols in K562 and K562/Adr cell types. Of special note, both cell types express different subsets of NFκB target genes. More particularly, K562 cells reveal a predominant inflammatory gene expression profile (i.e. strong expression of IL6, IL8, MCP1, and A1/Bfl1), whereas K562/Adr cells demonstrate a more tumorigenic pattern (i.e. strong expression of A20, cyclin D1, VEGF, and mdr1). As such, we further studied NFκB signaling mechanisms and coregulatory pathways which may be responsible for differential NFκB target gene expression/inhibition and apoptosis sensitivity for withaferin A and Siamois polyphenols. Upon characterization of the major NFκB activation and transactivation pathways, we found differential regulation of NFκB activity by withaferin A and quercetin, kaempferol, eriodictyol and WP283. Interestingly, IκB degradation and NFκB/DNA binding was significantly reduced by all compounds tested in both cell types, among which withaferin A, quercetin and eriodictyol showing the most potent inhibition, and kaempferol and WP283 much weaker and variable inhibition. Remarkably, increased levels of basal NFκB binding in K562/Adr cells cannot be inhibited by Siamois polyphenols in contrast to inhibition of inducible NFκB/DNA-binding. Furthermore, relative composition of NFκB/DNA binding complexes reveals that K562 cells contain much higher levels of p65-p65 homodimers. Of particular interest, the inflammatory cytokine IL8 was found to preferentially bind p65-p65 homodimers instead of p50-p50 and p50-p65 dimers [81], which could explain strong expression of inflammatory cytokines in K562 cells. From another perspective, NFκB dimer composition may also depend on the repertoire posttranslational modifications present on NFκB [63,70,71,82,83]. More specifically, we have detected variable and compound-specific effects on p38 MAPK, MEK1, Akt kinase pathways, which may also interfere with NFκB transcription factor composition and/or activity. Finally, besides phosphoregulation of transcription factors, acetylation by cofactors (CBP, HDAC, Sirtuin) and DNA methylation have recently added an additional epigenetic control of inducible NFκB transcription [84-87]. Of special note, as doxorubicin was found to increase Sirt1 HDAC levels [68], we compared nuclear Sirt1 levels in both cell types and observed a significant increase in Sirt1 protein in K562/Adr. As such, we cannot exclude that in addition to kinases also Sirt HDACs may contribute in cell-specific phosphoacetylation control of TF/DNA binding and transcriptional activity and may prevent NFκB p65 homodimer formation. In addition to cell specific regulation of NFκB, it can be observed from Fig. 5 that also AP1 members (i.e. Fra1, cjun and junD) and Nrf2 are differentially expressed in both cell types. As such, we can also neither exclude compound-specific kinase effects on these transcription factor families, since various NFκB target genes involved in inflammation, metastasis, angiogenesis and drug resistance are also coregulated by AP1 and Nrf2 [66,67,88,89].

Most surprisingly, although inhibition of NFκB activity in general contributes in chemosensitization of cancer cells [5,62], caspase activation is delayed and apoptosis is attenuated in K562/Adr cells treated with Siamois polyphenols, although efficacy of NFκB inhibition and initiation of early apoptosis by Siamois polyphenols is similar in doxorubicin-sensitive and resistant cell types. This is in line with previous reports on drug resistance, which describe that P-glycoprotein inhibits cytochrome c release and caspase-3/8 activation, but not formation of the death-inducing signal complex [23,24,90]. Along the same line, impaired activation of caspase 3,6,7,8,9,10 has been described in doxorubicin-resistant breast cancer cells [91]. The fact that Siamois polyphenols are able to completely ablate NFκB target gene expression, hyperactivate MEK1 and trigger early apoptosis in K562/Adr cells argues against the hypothesis that Siamois polyphenols may not be uptaken or are secreted out of the cell because of hyperactivated P-gp activity in K562/Adr cells. As such, P-gp overexpression confers resistance to a wide range of caspase-dependent apoptotic agents not only by removing drugs from the cell, but also by inhibiting the activation of proteases involved in apoptotic signaling [92]. Only a few drugs are reported to overcome this P-gp/Mdr phenotype and most of them are molecules that induce cell death in a caspase-independent manner [93]. Interestingly, in analogy to some specific glutathione S transferase inhibitors (NBDHEX) and mitochondria-targeting drugs (oligomycin) [94-96], withaferin A was found to bypass the P-gp resistance and to overcome attenuation of late apoptosis in K562/Adr cells. Unfortunately, we could not detect major differences in regulation of intracellular regulators of mitochondrial apoptosis of the Bcl2, BH123 or BH3 family proteins in K562 and K562/Adr cells treated with withaferin A or quercetin: both treatments trigger time-dependent decrease of Bcl2, Bim and P-Bad protein levels in K562 cells (albeit delayed in K562/Adr cells). However, upon investigation of cytoskeletal proteins, we observed that withaferin A is able to decrease tubulin protein levels, whereas Bcl_XL _and Bax protein levels remain unaffected. Interestingly, various chemoresistant tumors, including doxorubicin resistant cancers reveal therapy induced cytoskeletal changes in microtubules and intermediate filaments [97,98]. In analogy to other microtubule-targeted anti-cancer drugs, withaferin A could restore therapy sensitivity in P-gp-overexpressing cells by targeting the cytoskeletal organization. Further support for this mechanism has recently been provided by other groups, describing involvement of withaferin A-dependent actin and vimentin microfilament aggregation in cancer cell apoptosis and suppression of angiogenesis via a direct thiol oxidation mechanism [77,99,100]. Along the same line, we were able to block withaferin A-induced effects upon competition with excess amounts of the cysteine donor molecule DTT. Alternatively, it cannot be excluded that thiol-reactivity of withaferin A interferes with cysteine-sensitive P-gp protein folding steps and/or P-gp protein function [101,102]. Further investigation is needed to map cysteine target proteins of withaferin A which allow to bypass P-gp chemoresistance and restore apoptosis sensitivity.

## Conclusions

We found that transcriptional inhibition of NFκB-, AP1- and Nrf2- driven target genes involved in inflammation, metastasis, angiogenesis, drug resistance is not sufficient to overcome the P-gp-coupled attenuation of caspase-dependent apoptosis in K562/Adr cells. Remarkably, the withanolide withaferin A was found to relieve attenuation of caspase activation and apoptosis in K562/Adr cells, presumably via a direct thiol oxidation mechanism which targets cytoskeletal microfilaments, such as tubulin, actin and vimentin. This makes withaferin A an attractive natural phytochemical compound to overcome drug resistance and to elicit cell death in chemoresistant cell types. However, Siamois polyphenols could also have therapeutical benefit as well, upon suppression of cancer-promoting inflammatory cytokines and growth factors involved in cancer progression [103]. Furthermore, although less effective in immediate eradication of apoptosis-deficient tumor cells, chronic exposure to Siamois polyphenols may demonstrate significant long-term anti-cancer properties upon epigenetic modulation of P-gp function and cell survival [28,53,104-108]. The latter strategy may be beneficial to globally retard progression of aggressive refractory tumors, instead of chemotherapy of refractory tumors, which may further select for clonal expansion and evasion of chemoresistant and/or metastatic cancer cells.

## Abbreviations

NFκB: nuclear factor κB; IκB: inhibitory subunit of NFκB; IKK: IκB kinase; TNF: tumor necrosis factor; IL: interleukin; EMSA: electrophoretic mobility shift assay.

## Competing interests

The authors declare that they have no competing interests.

## Authors' contributions

WS carried out the molecular studies and drafted the manuscript. AP, SZ and SG assisted in molecular studies. SM, GH participated in the design of the study. WP has purified WP283. WVB conceived of the study, and participated in its design and coordination and helped to draft the manuscript. All authors read and approved the final manuscript.
